# Neutrophils from ANCA-associated vasculitis patients show an increased capacity to activate the complement system via the alternative pathway after ANCA stimulation

**DOI:** 10.1371/journal.pone.0218272

**Published:** 2019-06-19

**Authors:** Sophie Ohlsson, Lisa Holm, Christina Hansson, Susanne M. Ohlsson, Lena Gunnarsson, Åsa Pettersson, Lillemor Skattum

**Affiliations:** 1 Department of Nephrology, Institution of Clinical Sciences in Lund, Lund University, Lund, Sweden; 2 Department of Laboratory Medicine, Section of Microbiology, Immunology and Glycobiology, Lund University, Lund, Sweden; 3 Clinical Immunology and Transfusion Medicine, Region Skåne, Lund, Sweden; Hospital for Sick Children, CANADA

## Abstract

Anti-neutrophil cytoplasmic antibody (ANCA)-associated vasculitides (AAV), including granulomatosis with polyangiitis (GPA) and microscopic polyangiitis (MPA), are autoimmune conditions associated with small vessel inflammation. Earlier studies indicate that complement activation via the alternative pathway plays a major role in the pathogenesis. In this study we have investigated if ANCA-activation of neutrophils from AAV patients leads to activation of the alternative complement pathway. C5a-primed neutrophils (PMN) from 10 AAV patients and 10 healthy controls (HC) were stimulated with PMA or IgG purified from PR3-ANCA positive patients (ANCA IgG). The supernatants were analyzed for release of complement proteins and markers of different granules by ELISA, and release of microparticles (MP) by flow cytometry. The ability of the supernatants to activate the alternative complement pathway was determined by incubation with normal serum and C3bBbP and C5a were measured by ELISA. MP were analyzed by flow cytometry and removed by centrifugation. The supernatants from the AAV patients’ neutrophils produced significantly more C3bBbP compared with HCs (p = 0.0001). C3bBbP levels correlated with the number of MP. After removal of MP from the supernatants, alternative pathway activation was significantly lower. This study shows that primed and ANCA-stimulated neutrophils from AAV patients have a greater ability to activate the alternative complement pathway compared to primed neutrophils from healthy controls. This finding emphasizes the role of complement in the pathogenesis of AAV - underlining the therapeutic potential of C5a and other complement blockade.

## Introduction

Primary systemic vasculitis is characterized by relapsing-remitting inflammation and necrosis of blood vessel walls and sometimes granuloma formation. Small-vessel vasculitis lesions with little or no immune complex deposition (pauci-immune) in conjunction with anti-neutrophil cytoplasmic autoantibodies (ANCA) characterize ANCA-associated vasculitides (AAV). AAV affect small vessels in various organs, such as the kidneys, and include granulomatosis with polyangiitis (GPA) and microscopic polyangiitis (MPA). ANCA are in most cases directed against proteinase 3 (PR3) or myeloperoxidase (MPO), two important enzymes in the host defense against bacteria which are located in the granules of neutrophils and monocytes [1].

ANCA can activate primed neutrophils (PMN) to release their granular content, produce reactive oxygen species and mediate the release of microparticles (MP) from neutrophils as previously described by our group and others [2, 3]. In AAV, immune complexes and complement were previously considered not to be involved in the pathogenesis, since depositions detected by immunofluorescence are generally absent or scanty in the lesions, which differs from immune complex-mediated and anti-GBM glomerulonephritis [4]. However, low levels of immune complexes and complement do exist at sites of vascular inflammation and necrosis. Haas and Eustace performed studies on 126 renal biopsies from patients with crescentic glomerulonephritis associated with positive ANCA serology and/or necrotizing small vessel arteritis and found immune complex deposits in 54% of these [5]. In the majority of these cases immunofluorescence was positive for Ig and/or C3.

Activation of neutrophils is thought to play a major role in the pathogenesis and this was demonstrated *in vivo* when a murine disease model of MPO-ANCA vasculitis was established [6]. This opened new avenues for experiments that also suggested a critical role for complement activation in AAV, via the alternative and terminal pathways and that intervening in complement activation can prevent disease progression [7–9]. Anti-MPO IgG was induced in MPO-deficient mice and transferred into wild-type mice, resulting in crescentic glomerulonephritis. When the recipient mice were deficient in C5 or factor B of the alternative pathway no disease developed. Mice deficient in C4 developed glomerulonephritis to the same extent, indicating that there was no involvement of the classical or lectin pathways. When mice were pre-treated with a C5-inhibiting monoclonal antibody the lesions could be prevented.

After release from the bone marrow into the circulation, neutrophils can be primed by pro-inflammatory mediators, e.g. TNF-α and C5a and become attached to locally activated endothelium. ANCA can then activate these attached neutrophils. By mechanisms that are still unclear, the alternative pathway is activated, leading to generation of C5a which primes surrounding neutrophils by binding to C5a receptors. C5a recruits more neutrophils to the site through chemotaxis and creates an inflammatory amplification loop that finally results in necrotizing vascular injury [10]. Patients with ANCA disease produce higher plasma levels of complement factors C3a, C5a, soluble C5b-9, and Bb in active disease than in remission while no difference was reported in plasma C4d [8]. These data support the hypothesis that ANCA-induced neutrophil activation activates the alternative complement pathway.

Animal and in vitro studies have demonstrated a pivotal role of C5a and its neutrophil receptor C5aR (CD88). In a murine model of MPO-ANCA vasculitis, C5aR-deficient mice injected with anti-MPO IgG were protected from disease to a higher degree than wild type mice (5 out of 6) [11]. Further studies on blockade of the C5aR confirmed the central role in the pathogenesis of AAV and a clinical trial of a small-molecule C5aR antagonist is ongoing [12]. Less is known about the functional role of the second receptor of C5a, C5L2, and studies have shown contradictory results with anti- or pro-inflammatory effects in different disease settings [13]. The role of C5L2 in the pathogenesis of AAV was recently investigated in a study by Hao et al. Neutrophils where pre-incubated with a C5L2 blocking antibody, before C5a priming and subsequent stimulation with ANCA, leading to less release of granular proteins and less membrane expression of PR3—suggesting a pro-inflammatory role of C5L2 [14].

Clinical and experimental data strongly indicate activation of the alternative complement pathway, but the mechanisms by which complement activation occurs in AAV remain unclear. The current study investigated if supernatants from ANCA-activated AAV patient neutrophils activate the alternative complement pathway in normal serum and explored the routes of activation by investigating the expression of C5a receptors and the role of MP.

## Materials and methods

### Blood samples and patients

PMN were isolated from 10 PR3-ANCA positive patients in stable clinical remission, as assessed by Birmingham Vasculitis Activity Score (BVAS) 0 [15], and 10 healthy controls (HC). Table 1.

**Table 1:** PMN donor characteristics.

	Age Median (IQR)	Sex	Diagnosis	BVAS Median (IQR)	Corticosteroids (mg/day) Median (IQR)	ANCA
**AAV** (n = 10)	68 (15)	6/10 Male	10/10 GPA	0 (0)	5 (2.5)	10/10 PR3
**HC** (n = 10)	52 (32)	3/10 Male	-	-	-	-

PMN = polymorphonuclear neutrophils, AAV = ANCA-associated vasculitis, HC = healthy controls, IQR = inter quartile range, BVAS = Birmingham vasculitis activity score, GPA = granulomatosis with polyangiitis, ANCA = anti-neutrophil cytoplasmic antibodies.

Serum samples for IgG purification were obtained from AAV patients with PR3–ANCA specificity. The ANCA IgGs that we use have been well characterized before by toxicity index, epitope mapping and affinity [2]. HC IgG was used as negative control in some assays.

Written informed consent was retrieved from all donors and the Helsinki declaration was followed. These studies were conducted with permission from the Regional Ethical Review Board, Lund, Sweden, DNR 110/2008.

### IgG purification

IgG was purified using NAb protein G spin columns (Thermo Scientific, Rockford, IL, USA) and concentrated to 2 mg/mL, as previously described [2].

### PMN purification

Heparinized blood was collected from 10 patients and 10 HCs and the PMN were purified by density gradient centrifugation as previously described [2].

### Degranulation assay

We performed two series of experiments in which PMN were primed with TNF-α in one arm and with C5a in the other. PMN primed with TNF-α were stimulated with 10 different ANCA IgG as previously described by our group [2]. PMN primed with C5a were stimulated with 4 different ANCA IgG. After optimization experiments a C5a concentration of 60 ng/mL was chosen.

Purified PMN were adjusted to 5 x 10^6^ cells/mL in KRG (Krebs–Ringer buffer with glucose: 130 mM NaCl, 5 mM KCl, 1·27 mM MgSO4, 0·95 mM CaCl2, 10mM NaH2PO4/Na2HPO4, pH 7·4, 5 mM glucose). PMN were exposed to cytochalasin B (CLB, Sigma-Aldrich, Steinheim, Germany, 5 μg/ml) for 5 min followed by priming with TNF-α (R&D systems, Abingdon, UK, 2 ng/ml) or C5a (R&D Systems, Abingdon, UK, 60 ng/mL) for 15 min. The cells were then stimulated with IgG (200 μg/ml) for 15 min. All stimulations were performed at 37°C, with gentle shaking. As controls, cells stimulated with PMA (4 μg/ml), HC IgG or buffer only were used. Subsequent steps were performed as previously described [2].

### Degranulation ELISA

Supernatants and pellets from the degranulation assay were run through 5 different ELISAs to analyze the degree of degranulation, as previously described [16]. Albumin was used to detect release from secretory vesicles, gelatinase for gelatinase granules, lactoferrin for specific granules, myeloperoxidase for azurophil granules. The other main ANCA autoantigen PR3 was also analyzed. Subcellular fractionation of neutrophils has shown that PR3 can be detected mainly in azurophil granules, but also secretory vesicles and specific granules [17].

We also studied the release and total contents of properdin. Properdin was analyzed by the commercial Hycult Properdin ELISA kit from Nordic Biosite, with a detection rate of 0.31–20 ng/ml. Supernatants were diluted 1:25 and pellets 1:50.

Release was calculated as the amount of protein in the supernatant divided with the total amount of protein in the supernatant and pellet all together.

### ROS assay

Purified PMN were adjusted to 2x10^6^ cells/ml in Hanks’ balanced salt solution (HBSS++) (Hyclone) and primed with C5a 60 ng/mL for 15 min at 37°C followed by Cytochalasin B (Sigma-Aldrich, Steinheim, Germany; 5 μg/ml) exposure for 5 min at 37°C. PMN were then stimulated with either purified ANCA IgG (200 μg/ml), HC IgG or a monoclonal anti PR3 antibody (a kind gift from Dr Zhao, Beijing, 5 μg/ml), phorbol 12-myristate 13-acetate (PMA) (Glycotope Biotechnology, Heidelberg, Germany; 100 ng/ml) or HBSS at 37°C. This was performed in duplicate samples in white 96-well polystyrene plates (Thermo Scientific) in the presence of luminol (Sigma-Aldrich, Fluka; 1 μg/ml) and the scavenger superoxide dismutase (SOD) (Sigma-Aldrich; 5 U/ml) and catalase (Sigma-Aldrich; 2400 U/ml) to detect intracellular ROS, or isoluminol (Sigma-Aldrich; 1 μg/ml) alone to detect extracellular ROS. PMN were stimulated for a total of 60 min and luminescence, as a measure of ROS production, was monitored in a microplate reader (Tristar LB941; Berthold Technologies, Bad Wildbad, Germany) every 10 min. Each well was measured for 0·1 s with filter. The software MicroWin 2000 was used for analysis. We chose to report the value after 20 min of stimulation, as in most cases this time point corresponds to maximum stimulation.

### Complement activation assays by ELISA

The complement activation assays were performed on the PMN supernatants generated in the degranulation assay.

#### C3bBbP complex and C5a formation

100 μL of all supernatants from the degranulation assay were put on microdialysis against Veronal buffered saline (pH 7.4), MWCO 6–8000 at 4° overnight. Then 90 μL of microdialysis sample was added to 90 μL of HC serum on ice, with gentle shaking at 37°. 50 μL samples were removed at 0,1 and 4 hours and 5 μL 0.5 M EDTA was added. Samples were immediately put in the freezer at -80°C.

#### C3bBbP ELISA assay

Polystyrene 96-well plates (Maxisorp; Nunc, Roskilde, Denmark) were coated with anti-properdin IgG (IgG fraction of rabbit antiserum against human properdin, a kind gift from dr K Persson, dept. of Virology, Malmö) diluted 1/500 in PBS overnight at 4°. Plates were washed three times with ELISA wash (0.9% NaCl, 0.05% Tween 20) between each of the following incubation steps. ELISA plates were blocked with incubation buffer (PBS 1% BSA) while gently shaking for 2 hours at room temperature. Samples (1/10 in PBS-T (0.05% Tween 20)) were added for 2 hours at room temperature. The detection antibody a-C3c (IgG fraction of rabbit antiserum against human C3c, Dako A0062, Dako, Glostrup, Denmark) was conjugated with alkaline phosphatase and incubated at dilution 1/500 in PBS-T for 2 hours at room temperature [18].

#### C5a ELISA assay

C5a was measured in all samples (1/200) according to manufacturer’s instructions using a commercial C5a ELISA kit (A025 MicroVue, Quidel, San Diego, CA, USA).

### Detection, quantification and removal of MP

MP released from the stimulated cells were measured in the supernatants by flow cytometry (BD Accuri C6), as previously described [19]. Supernatants from the degranulation assay were used. In short, 100 μL supernatant was mixed with an equal volume of binding buffer and then incubated with 7 μL of CD66-PE (551480 BD Pharmingen) and 5 μL of Annexin V-FITC (556419 BD Bioscience) for 20 min at room temperature. The MP gate was determined by 0.8–1 μM fluorescent beads, as previously described. All CD66/Annexin V positive events identified by size were considered to be MP. Negative (50 μM EDTA in PBS) and positive (supernatants from PMN stimulated with 100 ng/ ml TNF-α) controls were included. MP were removed from the supernatants by centrifugation at 21 300 g, 30 min.

### Expression of C5aR1 and C5L2

Isolated and unstimulated PMN from 5 patients and 5 HCs were tested for C5aR and C5L2 surface expression by FACS. They were stained with monoclonal antibodies against C5aR, APC anti-human CD88 (C5aR) (BioLegend, San Diego, CA, USA) and C5L2, PE anti-human C5L2 (BioLegend, SanDiego, CA, USA) and analyzed using a fluorescence activated cell sorter (FACS) Canto II (BD Biosciences, Stockholm, Sweden). The samples were blocked using human IgG (0,5 mg/ml) in PBS containing 5% mouse serum and 1% BSA (Sigma Chemical Co., St Louis, MO, USA). The neutrophil population was identified by forward and side scatter gating. Mean fluorescence intensity (MFI) and percentage of C5aR and C5L2-positive cells were recorded.

The effects of priming with either TNF-α or C5a with or without pre priming with CLB were studied. A negative control was kept at 4°C. Samples were analyzed at 5, 10, 15, 30 and 60 min.

Further experiments were performed to test surface expression of C5aR and C5L2 at different time points on stimulated neutrophils from 2 patients and 2 HCs. PMN were primed with either CLB and TNF-α or CLB and C5a. Each sample was made in duplicates, one with only priming and one with the addition of a monoclonal

PR3 antibody (a kind gift from Dr Zhao, Beijing, 5μg/ml) at 15 min. Samples were then analyzed at 30 and 60 min.

### Statistical analysis

All statistical analyses were performed on GraphPad Prism 7.0 software (GraphPad Software, San Diego, CA, USA). Correlations were determined by Spearman’s correlation test. Mann-Whitney U-test was used for two group comparisons. All p-values were considered significant at p < 0.05.

## Results

### PMN stimulation

PMN from AAV patients and HC degranulated to a similar extent upon ANCA-stimulation after priming with TNF-α, as it has been shown previously [2] and the same was seen after priming with C5a. C5a priming, however, generated a significantly higher degree of degranulation, especially of PR3 and MPO, compared to TNF-α priming (45–50% versus 5–10%), Table 2. In order to obtain an extra effect, on top of the priming effect, with ANCA stimulation, the PMN had to be pre-incubated with CLB and there was a variation in the effect of individual ANCA IgGs. However, there was no significant difference when comparing to what degree the individual IgGs stimulated PMN from HC or from AAV patients. HC IgG also stimulated degranulation to some extent, Table 3.

**Table 2:** Degranulation (% release, median (IQR)).

PMN/Priming	HSA	MMP-9	LF	MPO	PR3	P
**AAV/TNF-**α (n = 50)	62 (9)	38 (22)	12 (12)	5 (5)	4 (4)	34 (22)
**AAV/C5a** (n = 20)	75 (8) p < 0.001	55 (21) p < 0.01	37 (15) p < 0.0001	44 (35) p < 0.0001	31 (18) p < 0.0001	44 (18) p = ns
**HC/TNF-**α (n = 50)	62 (13)	46 (17)	13 (6)	4 (3)	4 (3)	42 (6)
**HC/C5a** (n = 20)	75 (7) p < 0.001	55 (20) p < 0.01	40 (9) p < 0.0001	47 (14) p < 0.0001	42 (13) p < 0.0001	46 (12) p = ns

IQR = inter quartile range. PMN = polymorphonuclear neutrophils. HSA = human serum albumin, MMP-9 = matrix metalloproteinase 9 (gelatinase), LF = lactoferrin, MPO = myeloperoxidase, Pr3 = proteinase 3, P = properdin. AAV = ANCA-associated vasculitis. HC = healthy control. N = 50 AAV PMN samples (5 PMN donors, 10 different ANCA IgG stimuli) and 50 HC PMN samples (5 PMN donors, 10 different ANCA IgG stimuli) respectively. P-values representing significance of the differences between TNF-α priming and C5a priming. Statistical method: Mann Whitney.

**Table 3:** Degranulation controls (% release, median (IQR)).

Stimuli	HSA	MMP-9	LF	MPO	PR3
**HC IgG** (n = 20)	49 (8)	25 (6)	2 (3.4)	0.6 (0.5)	2 (1.6)
**PMA** (n = 10)	75 (5)	85 (22)	48 (9)	14 (29)	10 (17)
**PBS** (n = 10)	29 (5)	6 (2)	3 (3)	3 (3)	3 (3)

IQR = inter quartile range. PMN = polymorphonuclear neutrophils. HSA = human serum albumin, MMP-9 = matrix metalloproteinase 9 (gelatinase), LF = lactoferrin, MPO = myeloperoxidase, PR3 = proteinase 3, P = properdin. AAV = ANCA-associated vasculitis. HC = healthy control. PMA = phorbol myristate acetate. PBS = phosphate buffered saline. N = 20 samples = 10 PMN donors (5 AAV and 5 HC) and 2 HC IgG stimuli. N = 10 samples = 10 PMN donors (5 AAV and 5 HC).

Intracellular ROS production was measured by stimulating primed PMN with ANCA IgG in the presence of luminol together with the scavengers SOD and catalase. Extracellular ROS was measured using isoluminol instead, as it is not able to cross membranes. There was a substantial variation in the amount of intracellular and extracellular ROS obtained with different stimulating IgGs. Priming with C5a before ANCA IgG stimulation, generated increased intracellular ROS in AAV PMN compared to HC PMN (p<0,001), Table 4.

**Table 4:** Oxidative burst after ANCA stimulation.

PMN/Priming	IC ROS (RLU) median (IQR) p = AAV vs HC	EC ROS (RLU) median (IQR) p = AAV vs HC
**AAV/TNF**-α (n = 50)	10872 (5581) p = ns	1920 (1306) p = ns
**HC/TNF**-α (n = 50)	9589 (4405)	1942 (1211)
**AAV/C5a** (n = 20)	13080 (3440) p < 0.001	1580 (2819) p = ns
**HC/C5a** (n = 20)	7770 (2540)	980 (1963)
**PMN/Controls**		
**AAV/HBSS** (n = 10)	0 (143) p = ns	0 (44) p = ns
**HC/HBSS** (n = 10)	0 (0)	0 (6)
**AAV/PMA** **(n = 10)**	566 (9880) p = ns	336 (1374) p = ns
**HC/PMA** **(n = 10)**	8732 (5677)	956 (594)

PMN = polymorphonuclear neutrophils, IC ROS = intra cellular reactive oxygen species, EC = extra cellular, IQR = inter quartile range, AAV = ANCA associated vasculitis, HC = healthy controls, HBSS = buffer/negative control, PMA = phorbol 12-myristate 13-acetate, n = number of samples. P-values representing significance of the differences between AAV patients and HC. Statistical method: Mann Whitney.

### Complement activation and microparticle release

Alternative complement pathway activation was measured in the supernatants of stimulated AAV PMN and HC PMN. C3bBbP-formation as well as C5a levels were measured over time. C3bBbP-levels peaked at 60 minutes and C5a at 240 minutes and therefore those time points were chosen for further analyses.

**Fig 1:**
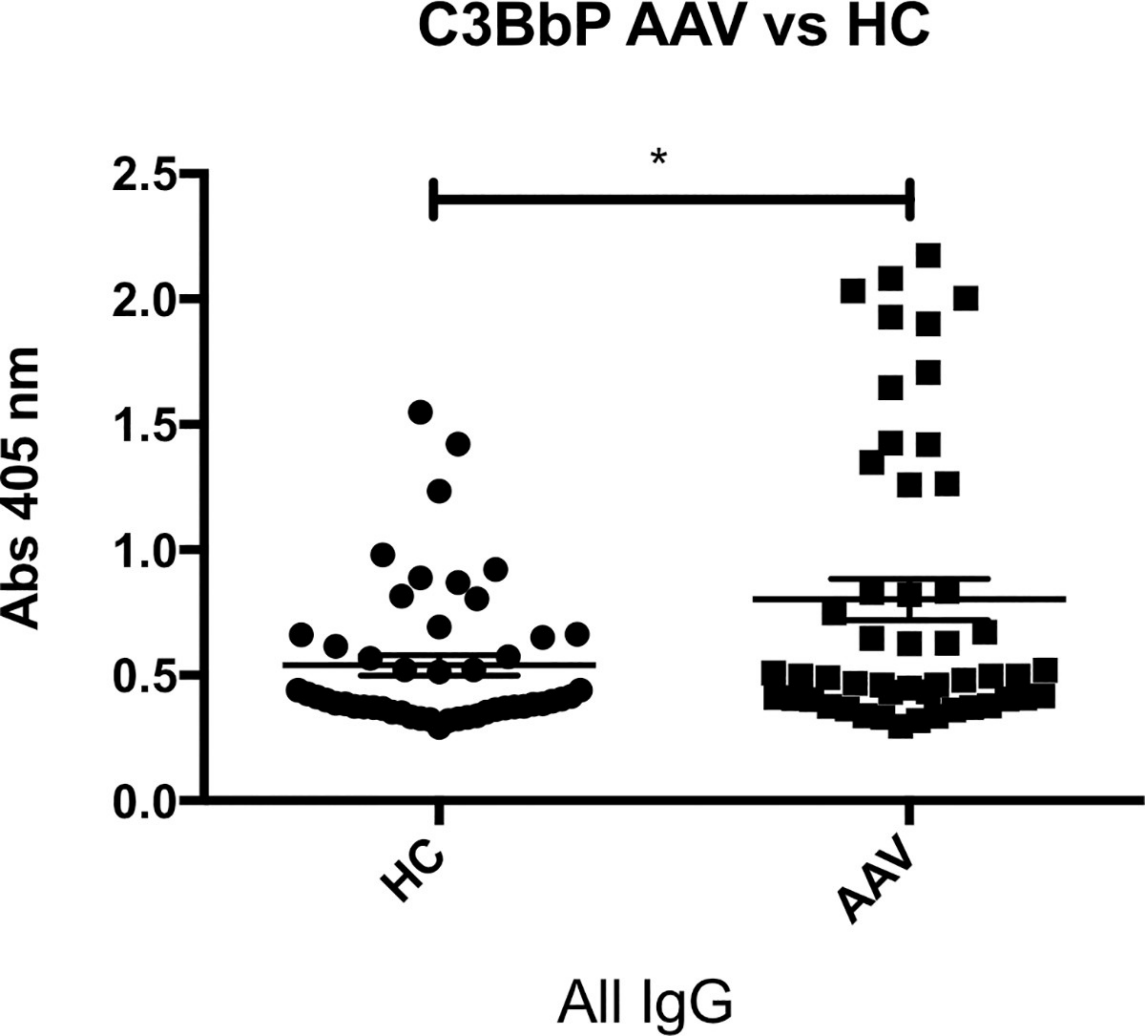
Increased C3bBbP levels in supernatants from AAV PMN. C3bBbP (stable alternative pathway C3 convertase, a sign of alternative pathway activation) formed in normal serum after incubation with supernatants from AAV PMN (neutrophils from vasculitis patients) or HC PMN (neutrophils from healthy controls), that have been primed with TNF-α and then stimulated with ANCA IgG (immunoglobulin G fraction from PR3-ANCA positive patients). Mean and SEM are shown. N = 50 AAV PMN samples (5 PMN donors, 10 different ANCA IgG stimuli) and 50 HC PMN samples (5 PMN donors, 10 different ANCA IgG stimuli). Statistical analysis: Mann Whitney, p = 0.01.

**Fig 2:**
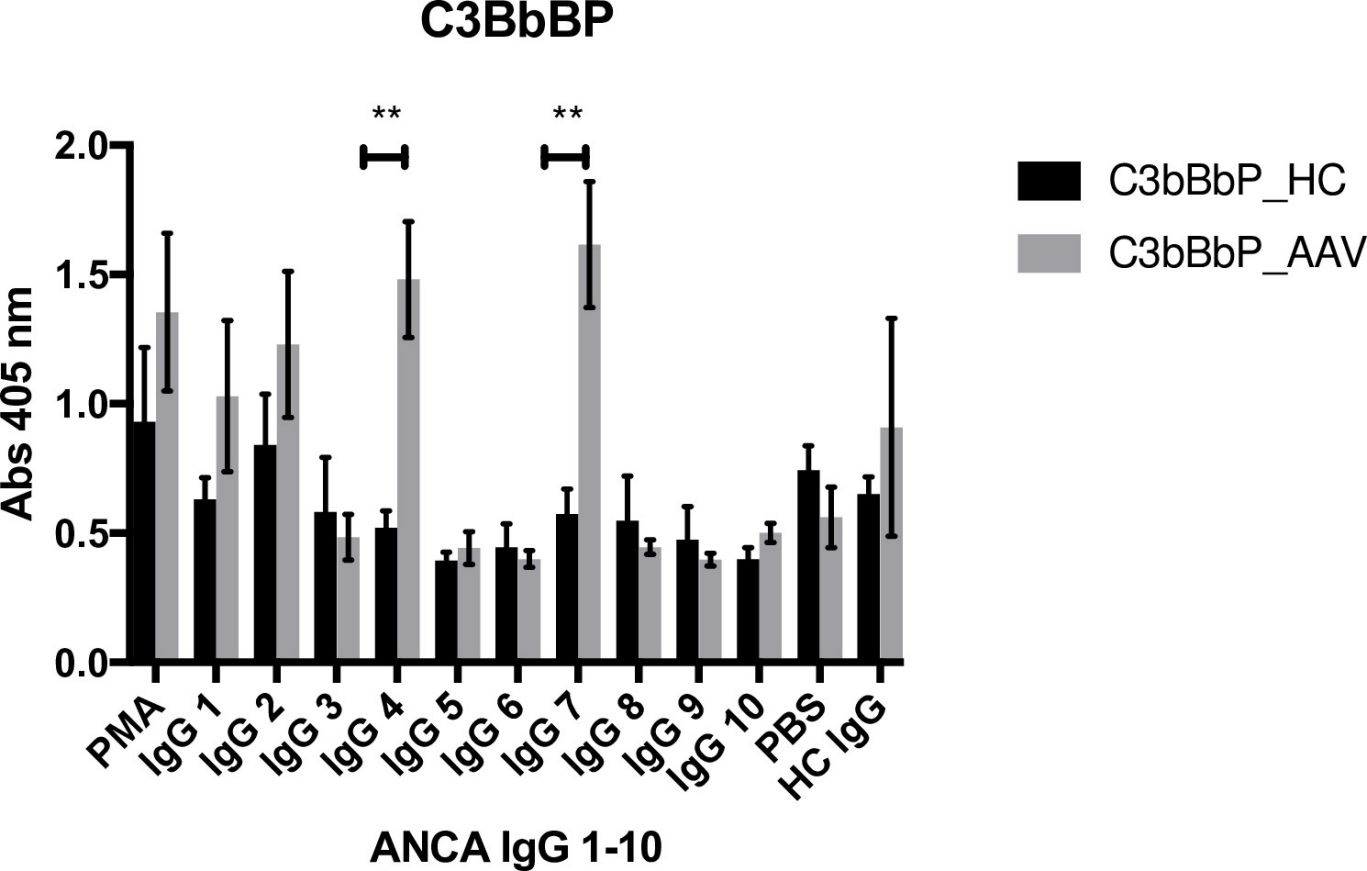
Activation with different ANCA IgG leads to varying degree of C3bBbP formation. C3bBbP (stable alternative pathway C3 convertase) formed in normal serum after incubation with supernatants from five AAV PMN (neutrophils from vasculitis patients) and five HC PMN (neutrophils from healthy controls) primed with TNF-α and then stimulated with 10 different ANCA IgGs (immunoglobulin G fraction from PR3-ANCA positive patients) and one HC IgG (immunoglobulin G fraction from a healthy blood donor). PMA (phorbol 12-myristate 13-acetate) was used as positive control and PBS as negative control. Number of neutrophil supernatants tested = 5 in each bar. Means and SEMs are shown. Statistical analysis: Mann Whitney, p < 0.001.

C3bBbP levels generated by incubation of the PMN supernatants with serum were significantly higher for AAV patients than for healthy controls, p = 0.01, Fig 1. There was a substantial variation in the amount of generated C3bBbP, depending on which ANCA IgG that had been used for PMN stimulation, Fig 2. Two of the ANCA IgGs having greatest impact (IgG4 and IgG7) had a significantly greater effect on AAV PMN compared to HC PMN, p < 0.001. Both Fig 1 and Fig 2 show results from PMN primed with TNF-α. Priming with C5a gave similar results, but with non-significant difference between patients and controls, possibly due to the strong impact by C5a itself on PMN and less additional effect of ANCA IgG stimulation.

**Fig 3:**
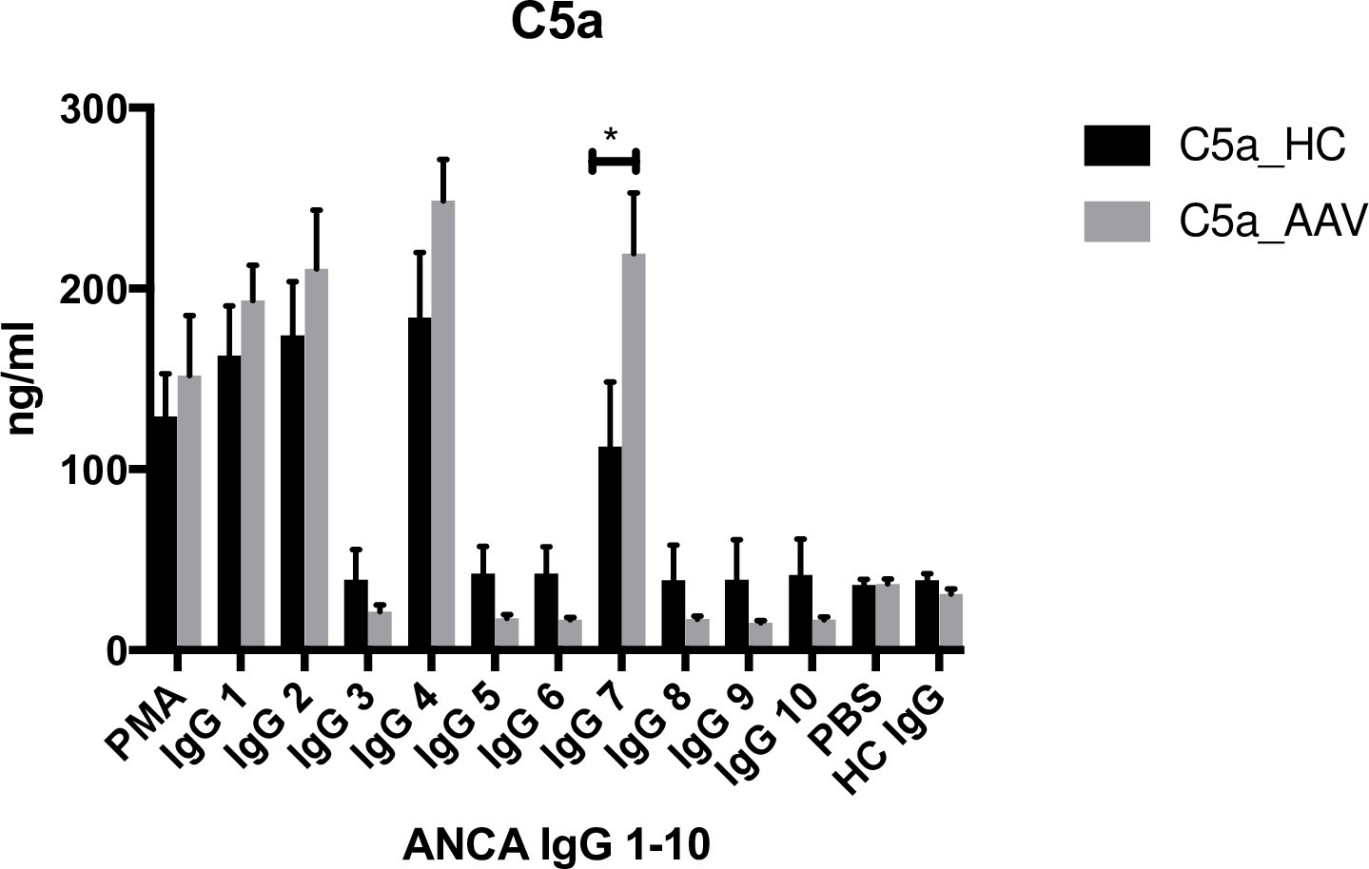
Activation with different ANCA IgG leads to varying degree of C5a formation. C5a measured in normal serum after incubation with supernatants from five AAV PMN (neutrophils from vasculitis patients) and five HC PMN (neutrophils from healthy controls) primed with TNF-α and then stimulated with 10 different ANCA IgGs (immunoglobulin G fraction from PR3-ANCA positive patients) and one HC IgG (immunoglobulin G fraction from a healthy blood donor). PMA (phorbol 12-myristate 13-acetate) was used as positive control and PBS as negative control. Number of neutrophil supernatants tested = 5 in each bar. Means and SEM are shown. Statistical analysis: Mann Whitney, p = 0.01.

There was a great variation in the amount of C5a generated with different stimulating ANCA IgGs, (Fig 3). One of the ANCA IgGs having greatest impact (IgG7) had a significantly greater effect on AAV PMN compared to HC PMN when priming with TNF-α, but no significant difference was seen between AAV patients and healthy controls when priming with C5a. C5a in the supernatants correlated with MP release (r = 0.3, p < 0.05).

Neither the neutrophil contents of properdin (median 15 ng/ml IQR 8 ng/ml, n = 10, vs median 17 ng/ml IQR 4 ng/ml, n = 10), nor the release upon neutrophil stimulation differed significantly between AAV patients and HC, Table 2. C3bBbP-generation correlated with C5a generation in both groups of PMN donors (r = 0,70 p<0,001) and with intracellular ROS in the AAV patients (r = 0.9 p < 0.001).

**Fig 4:**
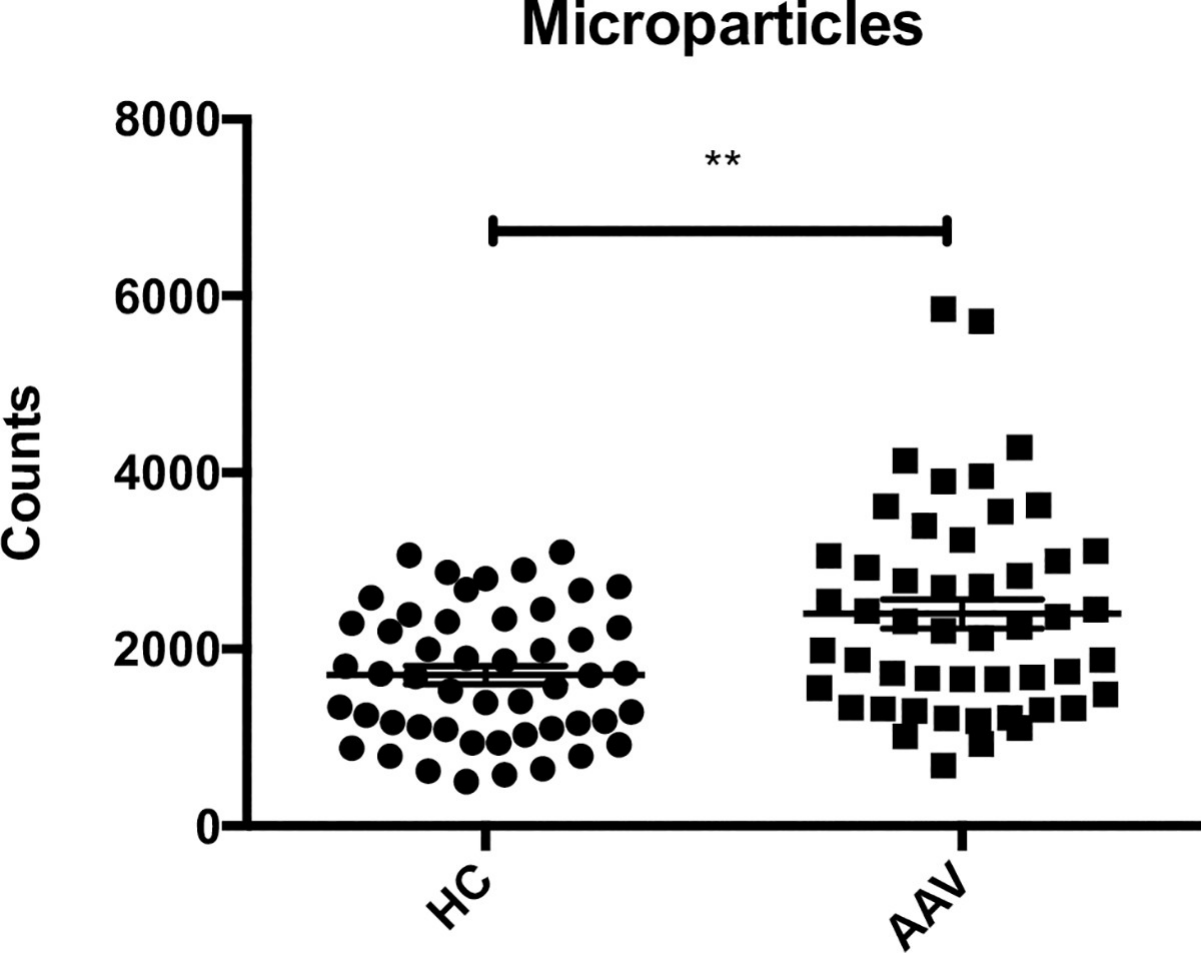
Increased microparticle release from AAV PMN. Release of CD66^+^ microparticles from five AAV PMN (neutrophils from PR3-ANCA positive vasculitis patients) and five HC PMN (neutrophils from healthy controls), after ANCA IgG (immunoglobulin G fraction from PR3-ANCA positive patients) stimulation, with 10 different ANCA IgGs. Means and SEM are shown. N = 50 AAV PMN samples and 50 HC PMN samples. Statistical analysis: Mann Whitney, p-value = 0.001.

**Fig 5:**
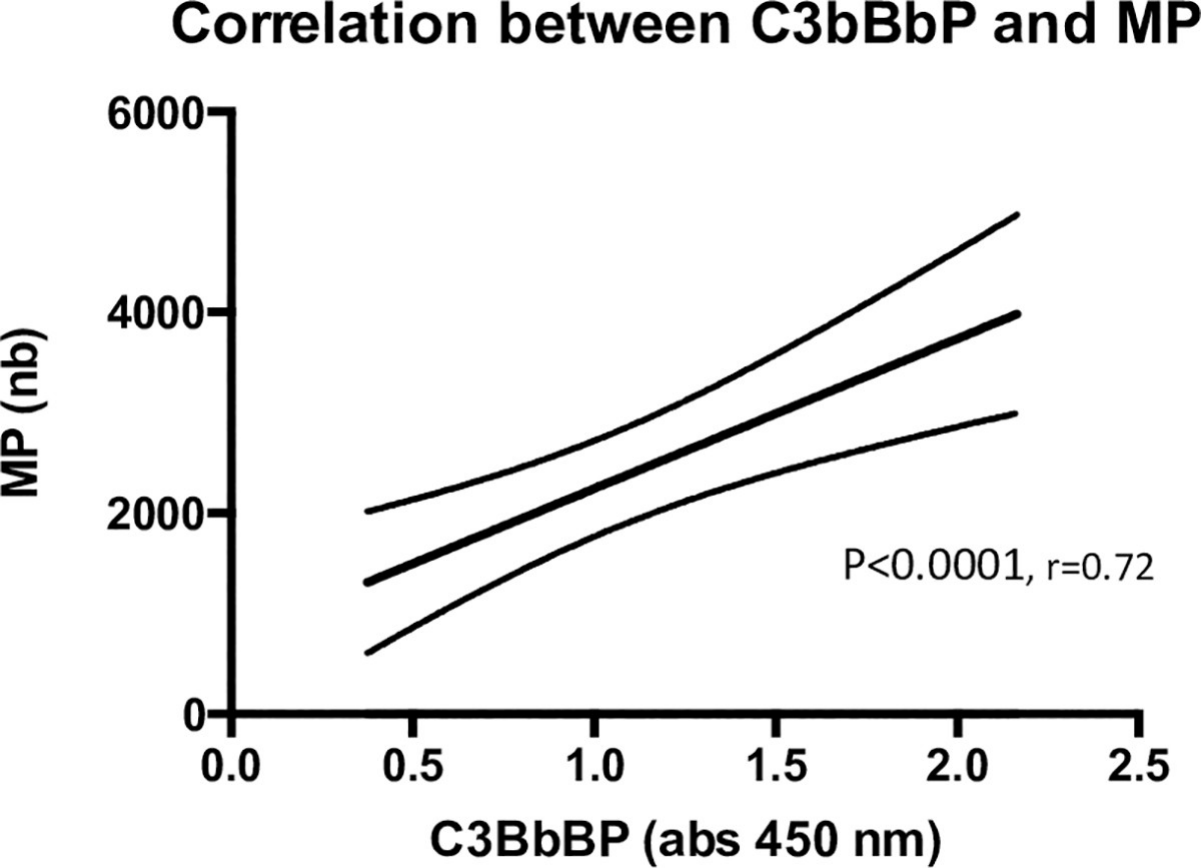
C3bBbP formation correlates with microparticle release. Correlation analysis of the number of CD66^+^ microparticles in PMN supernatants, and C3bBbP formation in normal serum, after incubation with the same supernatants. PMN supernatants were collected from 10 AAV PMN (neutrophils from 10 different PR3-ANCA positive vasculitis patients), after ANCA IgG stimulation, using two different ANCA IgG (IgG4 and IgG7 in Fig 1B), number of samples = 20, r = 0.72, p < 0,0001. Statistical analysis: Spearman.

**Fig 6:**
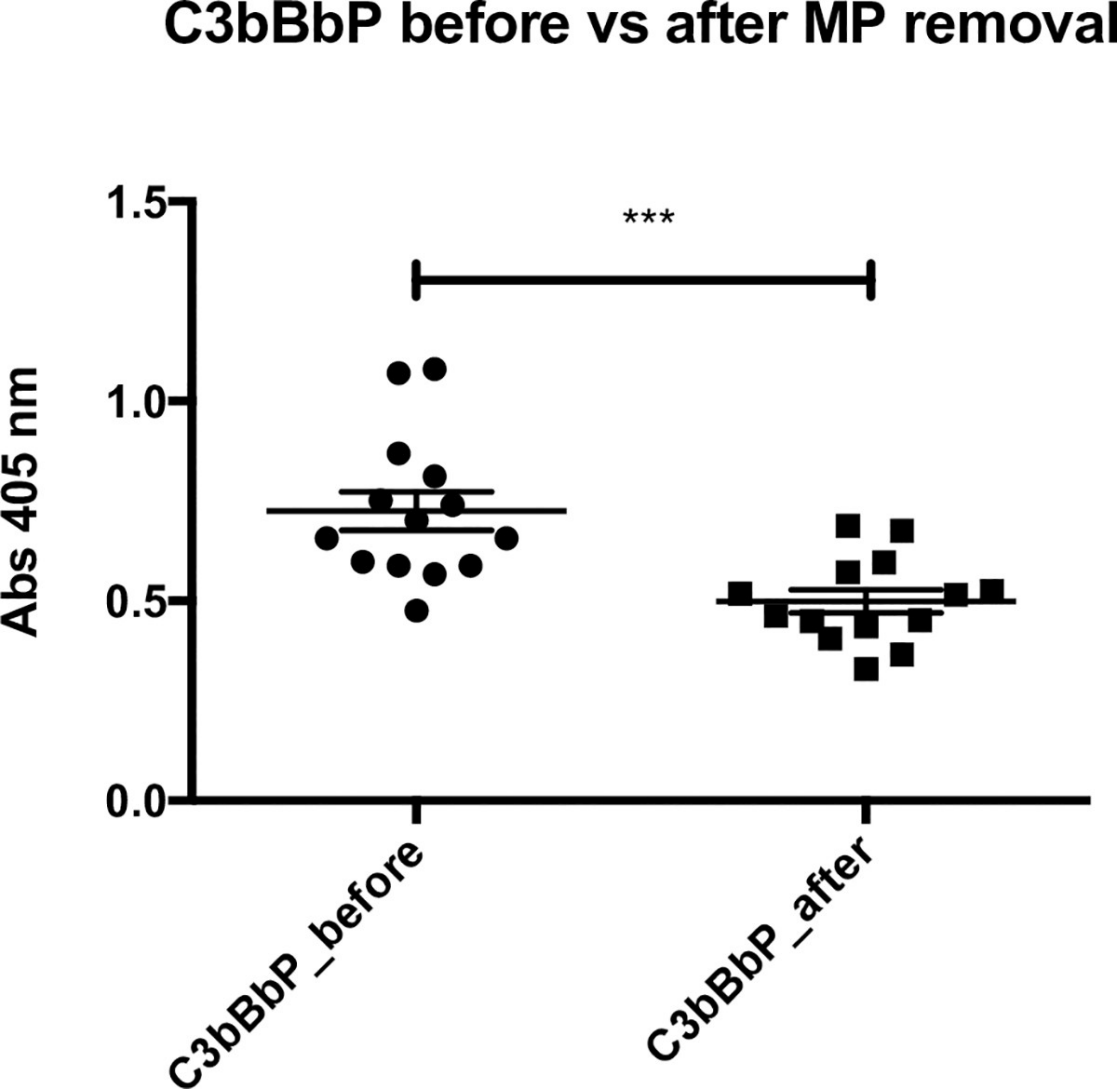
Less C3bBbP formation after microparticle removal. C3bBbP formation in normal serum after incubation with supernatants from seven AAV PMN (neutrophils from vasculitis patients) after ANCA IgG stimulation, using two different ANCA IgG (IgG4 and IgG7 in Fig 1B), before and after removal of CD66^+^ microparticles from the supernatants by centrifugation. Means and SEM are shown. Number of samples in each group = 14, p < 0,0001. Statistical analysis: Wilcoxon matched pairs signed rank test.

PMN microparticles were measured in the supernatants of the stimulated PMN. The released amount, after stimulation with ANCA IgG, was significantly higher in AAV samples compared with HC, Fig 4. There was a substantial variation in the amount of microparticles released, with different stimulating ANCA IgG. IgG7 had a significantly greater impact on MP generation from AAV PMN compared to HC PMN, p = 0.01. Supernatants from PMN only incubated with PBS contained very low MP levels (mean 662, SEM 79), whereas supernatants from PMN incubated with HC IgG contained slightly higher MP numbers (mean 1671, SEM 135). C3bBbP levels in the PMN supernatants correlated with the number of MP, regardless of priming with TNF-α (Fig 5) or C5a (r = 0.4, p < 0.001), and C3bBbP generation was significantly reduced when microparticles had been removed from the supernatants before incubation with healthy serum, Fig 6.

### C5a receptor cell surface expression

Since CLB was needed in order to get a significant effect of ANCA stimulation on degranulation and ROS-production after PMN priming with either TNF-α or C5a, we studied the effects of CLB and priming on PMN cell surface C5a receptor expression. Resting AAV and HC PMN displayed the expected C5aR/C5L2 distribution on their membrane and there was no difference between AAV patients and HC (data not shown).

**Fig 7:**
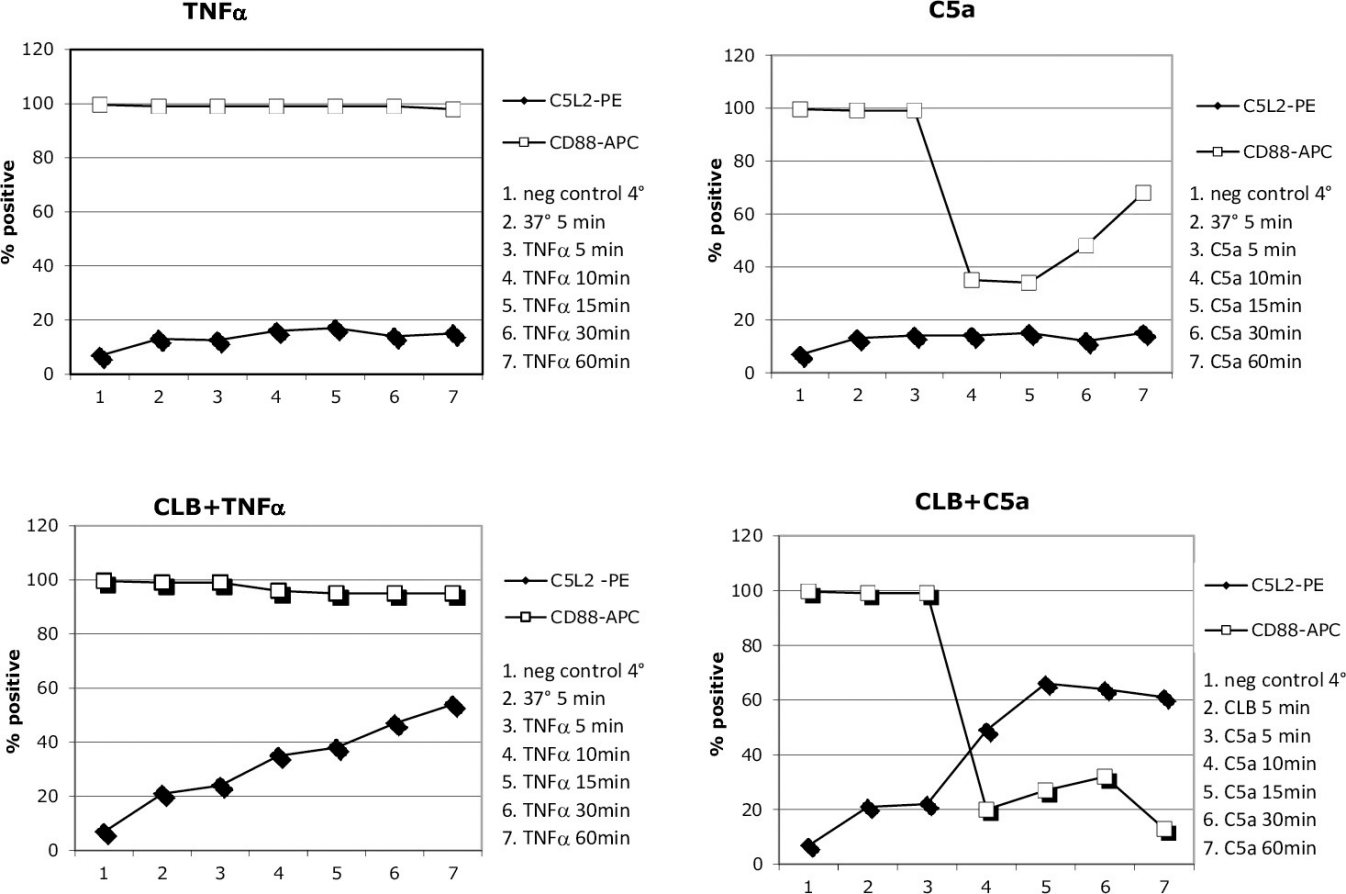
Effects of priming on membrane C5aR expression. Effects of pre-priming with CLB (cytochalasin B) or not, in combination with TNF-α or C5a priming, on membrane C5aR expression on HC PMN (neutrophils from healthy controls). Percentages of cells expressing CD88 and C5L2 are shown. Number of experiments = 2, showing representative values.

TNF-α itself did not seem to have any significant impact on C5a receptor expression. C5a seemed to induced temporary CD88 down regulation/internalization. CLB pre priming increased and prolonged CD88 down regulation and led to reciprocal C5L2 up regulation, Fig 7.

**Fig 8:**
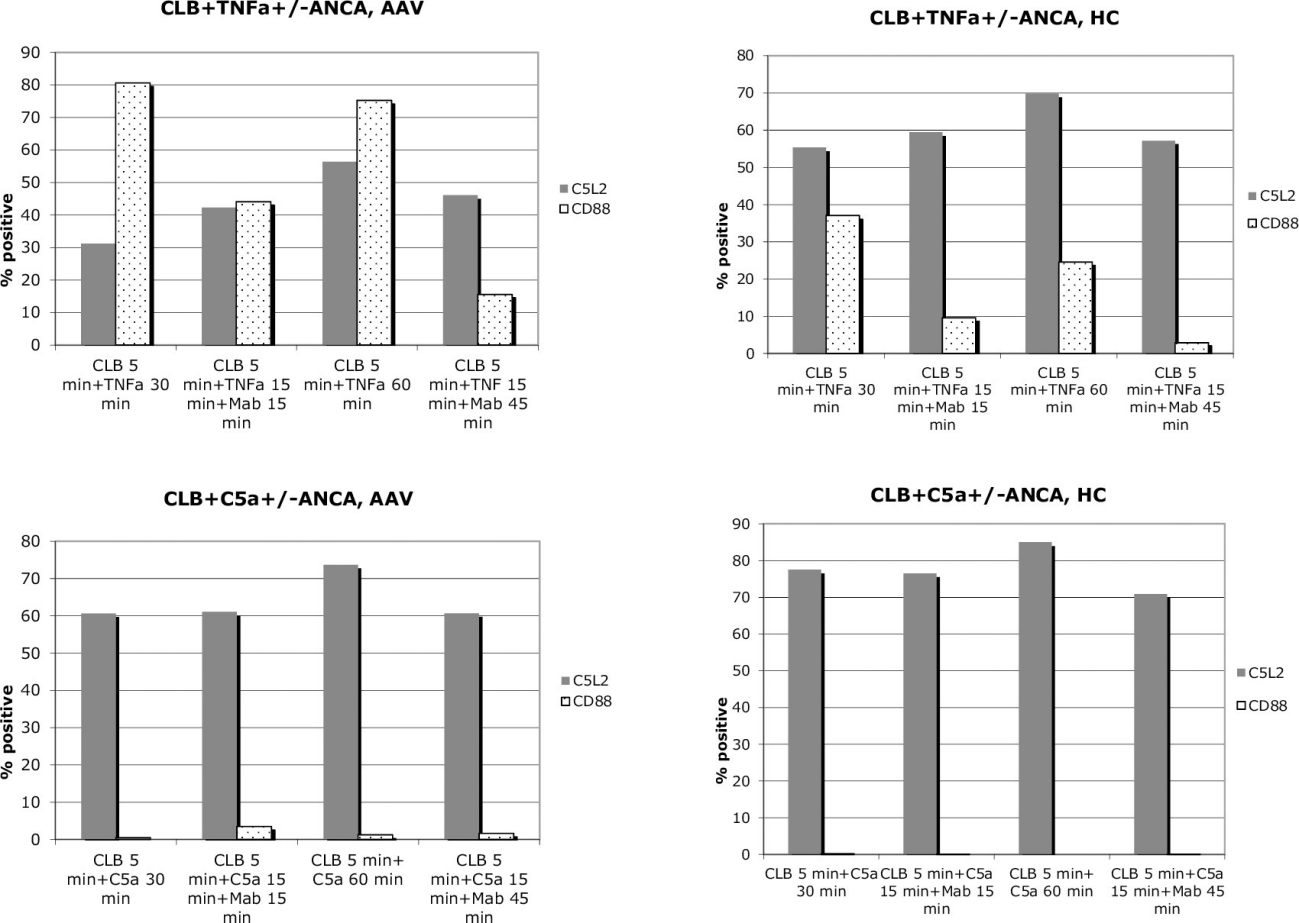
Effects of priming and ANCA IgG stimulation on membrane C5aR expression. Effects of ANCA IgG stimulation of primed AAV PMN (neutrophils from two different vasculitis patients) on membrane C5aR expression are displayed to the left and HC PMN (neutrophils from two different healthy controls) are displayed to the right. Percentages of cells expressing CD88 and C5L2 are shown. Number of experiments = 2, showing representative values.

We also studied the additional effect of adding ANCA IgG, Fig 8. Stimulation of PMN from AAV patients and HC with ANCA IgG, after priming with either TNF-α or C5a was performed. AAV PMN tended to downregulate CD88 less efficiently than HC PMN, however the experiments were only done on PMN from 2 AAV patients and 2 HC.

## Discussion

The alternative complement pathway has received increasing attention in the context of AAV during the last 10 years. Both animal studies and a clinical trial on C5aR blockade have given us evidence of its importance in these traditionally pauci immune diseases. [9, 11–13] Previous studies have predominantly focused on MPO-ANCA-associated vasculitis and stimulation with MPO-ANCA, probably since the existing animal models are in MPO-ANCA associated disease. In this study we studied patients with PR3-ANCA-associated vasculitis and PR3-ANCA IgG preparations. Due to results from other studies, we focused on the potential role of the alternative pathway although our findings do not exclude involvement of other complement activation pathways. We could demonstrate increased C3bBbP generation in supernatants from AAV PMN compared with healthy controls. This effect correlated with intracellular ROS production and microparticle release, whereas no correlation could be seen neither with simply the degree of degranulation, nor with release of properdin or PMN contents of properdin. These findings imply a loss of inhibition in AAV PMN. Deficient function of factor H has recently been described in AAV by Chen et al, leading to decreased alternative pathway regulation [20–22]. Decreased regulation of the alternative pathway in the fluid phase would be expected to lead to increased production of anaphylatoxins, creating a vicious circle of increased complement activation and increased PMN activation, already suggested in the pathogenesis of AAV[23]. In our degranulation assay, priming with C5a instead of TNF-α resulted in a 10-fold increased release of the ANCA antigens PR3 and MPO, implying an increased exposure and thereby possibly an increased risk of autoimmunity in C5a-driven inflammation.

Intracellular ROS production was found to be increased in AAV PMN, which is consistent with our previous results–and this has also recently been confirmed by Nishide et al, describing a possible relationship with low levels of membrane bound SEMA4D, leading to loss of inhibition.[24] Interestingly, we as well as others, have seen that the results of in vitro stimulation of PMN with ANCA, are largely dependent on the use of CLB, a compound known to inhibit actin polymerization, and thereby affecting several processes such as phagocytosis, pinocytosis, membrane transport of glucose and nucleosides, and perhaps also the distribution of available receptors on the cell surface [25–28]. The C5a receptor C5L2 is normally internalized by clathrin-mediated endocytosis, but after CLB treatment C5L2 expression on the cell surface increases, Fig 7. When exposing the PMN for C5a, CD88 is internalized as expected, however the effect is enhanced and prolonged by CLB pretreatment. Is there a circulating factor in vivo, affecting the PMN in a fashion similar to CLB? One candidate is Sphingosin-1 phosphate, which has some similar effects and has been implicated to play a role in AAV, and is known to have a role in the CD88/C5L2 balance [29, 30]. The C5L2 function has been debated, but there is increasing evidence of a pro inflammatory function and Hao et al could demonstrate reduced PMN priming for oxidative burst and degranulation by ANCA stimulation, after C5L2 blockade [14, 31]. Increased C5L2 expression has been seen in renal biopsies from AAV patients. [32] There is an ongoing clinical trial on C5aR blockade in the treatment of AAV [12]. The compound under study is selective for CD88 and it is unclear how selective CD88 blockade will effect C5L2 expression, which thus would be interesting to study.

Camous et al demonstrated that PMN-derived microparticles could activate the alternative complement pathway. [33] Our results point in the same direction, given the correlation between microparticle release and C3bBbP formation, and the demonstration of increased microparticle release from AAV PMN compared to HC PMN, after stimulation. In addition, AAV PMN seem to respond to stimulation in a way leading to more alternative pathway activation, compared to HC PMN. The mechanisms behind these findings need to be further explored. A role for properdin in the complement activation, as was hypothesized also in the article by Camous, cannot be excluded although we found no difference in neutrophil properdin content or expression between cells from patients and controls. Expression of properdin and other complement proteins on neutrophil-derived microparticles would be an important focus of further studies. A possible limitation to our study is the definition of microparticles within 0.8–1 μm, excluding smaller microparticles that might be biologically important [34], and this will be taken into consideration in future studies.

## Conclusions

This study shows that supernatants from stimulated AAV PMN have a greater ability to activate the alternative complement pathway compared to HC PMN. This increased ability could be caused by increased release of microparticles. Our findings emphasize the role of complement in the pathogenesis in AAV - underlining the therapeutic potential of C5a and other complement blockade.

## Abbreviations

ANCA: anti neutrophil cytoplasmic antibodies
AAV: ANCA-associated vasculitis
PR3: proteinase 3
MPO: myeloperoxidase
GPA: granulomatosis with polyangiitis
HC: healthy controls
PMN: neutrophil granulocytes
MP: microparticles
MPA: microscopic polyangiitis
ELISA: enzyme-linked immunosorbent assay
PMA: phorbol myristate acetate
IgG: immunoglobulin G
C5a: complement factor 5a
TNF-α: tumor necrosis factor alpha
FACS: fluorescence activated cell sorting
C5aR: complement factor 5a receptor, CD88
C5L2: complement factor 5a receptor 2
BVAS: Birmingham vasculitis activity score
ANCA IgG: Immunoglobulin G fraction from PR3-ANCA positive patients
CLB: cytochalasin B
